# New Antibiotics—Resistance Is Futile

**DOI:** 10.1371/journal.pbio.0020053

**Published:** 2004-02-17

**Authors:** Tabitha M Powledge

## Abstract

It is the certain fate of all antibacterials to be fought off eventually by the pathogens they target. We need new ways to defeat disease, and we will need them forever

By next summer, more than 40% of Streptococcus pneumoniae strains in the United States will resist both penicillin and erythromycin, according to a recent prediction from the Harvard School of Public Health. The forecast, based on mathematical modeling, was published in the spring of 2003. It's too early to tell whether that prediction is precisely on track, according to the senior author on that paper, Marc Lipsitch. But no one doubts that multidrug resistance in this common bug—responsible for diseases that range from sinus trouble and ear infections to meningitis and pneumonia—is speeding up.

It is the certain fate of all antibacterials to be fought off eventually by the pathogens they target. The fact that the process is accelerating has been alarming public health officials for some time, especially in the United States. We need new ways to defeat disease, and we will need them forever.

## Tried and True—and Tired?

Antibiotics have traditionally been plucked from nature's battleground. For billions of years, tiny organisms have engaged in an arms race, hurling toxic molecules at each other in the struggle to prosper. Nearly all of today's antibiotics are versions of weapons long wielded by microbes and fungi. Chemical synthesis of entirely human-created antibiotics has so far yielded only fluoroquinolones, a group of broad-spectrum antibiotics that includes Cipro, which became famously scarce during the 2001 anthrax scare, and linezolid (trade-named Zyvox), which is effective against some resistant strains of Staphylococcus, Streptococcus, and Enterococcus.

The usual way to find a new antibiotic has been laborious screening of immense libraries of compounds, natural and otherwise. Some argue that screening chemical libraries is approaching a deadend. There may be diminishing returns from screening, but it's not quite dead yet: in October, researchers at the University of Wisconsin at Madison reported a new class of bacterial RNA polymerase inhibitors with antibiotic potential. They were found by screening for molecules that prevent Escherichia coli from transcribing RNA.

Christopher T. Walsh of Harvard Medical School says screening's problem may be simply that libraries aren't good enough. Marine organisms have not been studied well, he points out, and 90% of organisms in the biosphere can't be cultured in standard ways. He says, “We're missing 90% of them every time we go and look in nature.”

Walsh is doing his bit to create new libraries. He and his colleagues have recently employed combinatorial biosynthesis to learn how to use part of the machinery for assembling cyclic peptide antibiotics to control their architecture. The result was a small library of natural product analogs, some of which have improved antibiotic activity against common bacterial pathogens. “There are dozens of such enzymatic domains that in principle one could clone, express, and test with other substrates. I view that as the kind of thing we should do,” he says. For example, Walsh suggests, it is a reasonable approach to second-generation improvement of daptomycin, the antibiotic most recently approved for sale in the United States.

## Improving on Nature

**Figure 1:**
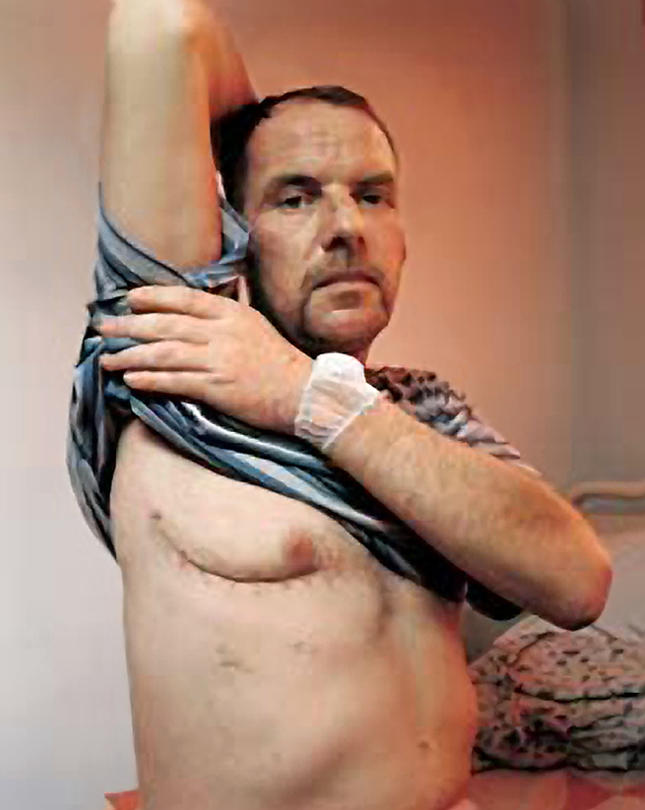
TB Drug Resistance This 40-year-old Estonian truck driver's TB is resistant to drugs and his right lung was removed three days before this picture was taken. (Photograph by WHO/STB/Colors Magazine/J. Langvad.)

Walsh collaborates with Chaitan Khosla of Stanford University on finding ways to make existing antibiotics better. They are studying biosynthesis of rifamycin, an antibiotic that is increasingly less effective against its prime target, tuberculosis (TB) (see Figure 1). “In the course of learning about that pathway, we've learned a few interesting things lately about how that molecule is initiated, and we're trying to apply it in other contexts, especially in the context of erythromycin biosynthesis,” Khosla says. The idea would be to make a molecule that might be more effective against bacteria that are becoming resistant to rifamycin—and are already naturally resistant to molecules like erythromycin.

“Basically, what we do is to try and figure out new ways to hijack the biosynthesis of antibiotics in nature so as to modify their structures with the goal of improving them,” Khosla explains. He works with an important class of natural antibiotics called polyketides that have generated dozens of drugs, including erythromycin.

Polyketides are secondary metabolites (which give their producers a competitive advantage in their environment) produced mostly by bacteria and fungi and made by a complex and structurally diverse family of enzymes called polyketide synthases (see the primer by David Hopwood in this issue of *PLoS Biology*). Among them are the anthracyclines, a group of anticancer drugs and antibacterials that includes tetracycline. In this issue of *PloS Biology*, Khosla and his colleagues report that they can make selective positional modifications in existing anthracycline antibiotics by starting in a different way with a different starting molecule. The molecule came from a natural anthracycline antibiotic, an estrogen receptor antagonist called R1128. R1128 is made via two modules of enzymes that work sequentially; the first module starts the process, and the second completes it. This division of labor permitted the researchers to tack the first R1128 module onto two other enzyme systems, thus engineering completely new anthracyclines. Some were more active in two types of assays than the natural parent molecule. “One setting was an assay on an estrogen-sensitive cancer cell line. Another setting was an assay to probe activity of an enzyme that's of particular interest in Type 2 diabetes, called glucose-6-phosphate translocase.” The work also revealed fundamental mechanistic features of the polyketide synthases, Khosla says.

The researchers didn't study the new anthracyclines' effects on bacteria, but Khosla notes that the general principle should apply to other classes of compounds, although the details of how it's implemented will vary from system to system. He says, “The upshot of this paper is that it is now possible to modify a particular methyl group in just about any anthracycline antibiotic.”

## Finding New Targets

Instead of searching for new antibiotics by modifying existing ones, some researchers are trying something completely different—first finding the most vulnerable targets in a bacterium and then designing something that hits one or more of them hard. “You have to understand a helluva lot more about how these little cells work. In fact, we think we understand a lot, but I think we can understand almost everything now that we have all the genomes,” says Lucy Shapiro of Stanford University School of Medicine. While having full genome sequences—more than 100 microbe sequences have been completed—is essential, Shapiro believes that knocking outs genes galore to find out which ones are necessary and going after them all is not a sensible strategy. She observes, “People have been doing that for a while with absolutely no success. That's really going after the problem with a Howitzer instead of with an intelligent approach.”

So instead of screening libraries of existing compounds, Shapiro prefers using structural information about drug targets or their natural ligands to create new drugs, an approach known as rational drug design. And instead of looking at all essential genes in a bacterium and choosing one to target, she and her colleagues look at genetic circuitry that controls the cell cycle, the pathway that coordinates cell growth and differentiation. They have identified key control points, or nodes, in the circuitry for their favorite study subject, Caulobacter crescentus. Thus, they have found critical genes encoding proteins that control several critical functions in the cell. Their first candidate was an essential enzyme, a methyltransferase called CcrM, that prevents a particular piece of DNA from being expressed in a cell by tagging it with a methyl group.

Antibiotic discovery is all chemistry, Shapiro says, which is why she joined with biochemist Stephen J. Benkovic of Pennsylvania State University. They didn't know the structure of CcrM, Benkovic explains, but the literature about other methyltransferases suggested that the adenine molecule, which is the substrate for CcrM within DNA, binds to a specific region of the enzyme.

The researchers designed adenine-like molecules that would bind to CcrM and then developed inhibitors. Benkovic says, “We already knew what kind of structure we wanted, and we simply fine-tuned it.” They worked their way through 1,000 inhibitor candidates, ending up with a small subset—no more than about 20—that not only inhibited CcrM, but also killed Caulobacter very quickly.

And not only inoffensive Caulobacter. The compounds knock out other gram-negative bacteria, such as the pathogens Brucella abortus and Francisella tularensis. Some even killed off anthrax, a big surprise because it is gram-positive and so has much thicker cell walls than gram-negative bacteria. The researchers undertook an exhaustive series of experiments to identify which gram-positive bacteria would be affected by which compounds. The list of sensitive pathogens now includes multidrug-resistant Streptococcus, Staphylococcus, and Mycobacterium tuberculosis.

More recently, Shapiro reports, they have demonstrated efficacy against rats infected with anthrax or multidrug-resistant Staph, although the compounds save only about 60% of the rats at present. She notes, “So we have a long way to go. But this has proven that if you go after something using some rational approach instead of hit-and-miss, you'll probably have more success than by the other method.”

Benkovic points out that theirs is an entirely new class of compounds, small molecular weight compounds that can be made in a few steps. He says, “They don't look like the normal antibiotic, so that's why I think they're fairly unique.” The basic research was done under a grant from the Defense Advanced Research Projects Agency (DARPA), the United States Department of Defense's (DOD) central research and development organization, and once the researchers realized they wanted to develop drugs against three agents that have been considered bioterrorism threats — Brucella, tularensis, and anthrax — they established a separate operation, Anacor Pharmaceuticals, which is developing them with DOD funding and without Shapiro. In her Stanford lab, she continues her fundamental research to define the complete genetic circuitry of Caulobacter, hoping to identify additional nodes in the circuit. She says, “I am not doing it to develop antibiotics; that's what comes out of the work. My goal is to understand how the cell works. I think a lot of studies in pathogenesis should not be just to understand pathogenic organisms, but to understand the complete network of regulatory mechanisms that controls the bacterial cell.”

## Phage Therapy

**Figure 2:**
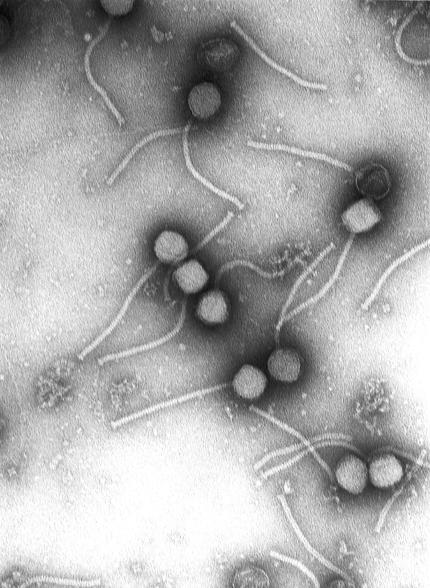
Phage Negative stain electron micrograph of the gamma phage from which the PlyG lytic enzyme was cloned for use to control B. anthracis. (Photograph courtesy of Vincent Fischetti and Raymond Schuch, The Rockefeller University.)

The most radical approach to new antibiotics may be the resurrection of an old idea: bacteriophage therapy (see Figure 2). Late in the 19th century, a researcher noticed that water from some of India's sacred rivers combated cholera. Some years later, the active agents were identified as viruses that infected bacteria. Such viruses are called bacteriophage, or phage for short. There were reports of phage success against dysentery, typhoid, and plague, and bacteriophage therapy had a brief heyday, especially in the 1920s. Results on other diseases were mixed, and with the appearance of antibiotics, phage therapy became unfashionable in the United States, although it has continued in Russia and Eastern Europe.

Phage were the model organisms of choice for genetics research in the 1930s and 1940s, but became less fashionable as research tools when investigators moved on to eukaryotes. A few held on, like Ry Young of Texas A&M University, who has made phage-induced cell lysis his life's work. “The cell is basically genetically dead as soon as the phage goes in there, but it will keep living as sort of an infected zombie for as long as the phage wants it to, with virus particles accumulating inside the cell,” he explains. “Only when the phage is ready and has decided that it's the right time will it pull the trigger. And the cell blows up.” The freed phage then spew forth to infect new cells.

Antibiotic resistance has led to new interest in phage therapy by several small biotech companies. Young continues basic research at Texas A&M, but has also joined one of them, GangaGen, providing bacteriophage expertise to its labs.

Phage do kill pathogenic bacteria effectively, and they do it without penetrating human cells, which they can't even recognize. So what is keeping phage therapy out of the clinic? Problems that some doubt can be overcome.

Because bacteria develop resistance to phage rapidly, phage therapy companies will need to direct cocktails against a single pathogen, according to Vincent Fischetti at The Rockefeller University. Phage are also antigenic, and the antibodies they stimulate will neutralize their effects during subsequent treatment, he says. But the chief problem appears to be regulatory—regulatory in the political, rather than the genetic, sense. When bacteriophage package their DNA, they occasionally include varying amounts of their hosts' DNA, too. This miscellany, Fischetti points out, is likely to make the Food and Drug Administration unhappy. “Phage normally are very fragile, their tails break, so lot-to-lot homogeneity could be a problem too,” he adds. “So even though it will work, I think they'll have an uphill battle.” Phage may well enter agricultural or veterinary use, he predicts, but are probably not going to be available to patients in the United States any time soon.

Fischetti chose a different approach to phage therapy. It does not rely on phage themselves, but on enzymes that phage produce to smash their way out of their host bacteria so they can infect new hosts. He and his colleagues employ these enzymes externally to kill bacteria. He reports, “We now have enzymes that will kill Strep pyogenes, pneumococci, Strep pneumoniae, Bacillus anthracis, Enterococcus faecalis, and group B Strep. The beauty of these enzymes is that they are targeted killing. You only kill the organism you intend to kill, without destroying or affecting the surrounding organisms that are necessary for health.”

The enzymes can be loaded into a nasal spray that wipes out pathogens such as Pneumococcus, Staphylococcus, and group A Strep on contact with mucous membranes. The strategy might prevent bacterial infections from spreading in close quarters like hospitals, nursing homes, and daycare centers. Fischetti says, “Clinical trials would tell us how often we had to treat, but more important, we'd have a reagent that could treat people who walk out the door of the hospital to eliminate or reduce the transmission of resistant organisms into the community. We don't have that capability right now.”

Fischetti and his colleagues have moved on to using the enzymes systemically to wipe out Bacillus anthracis spores, preventing them from germinating and seething through the bloodstream, producing deadly toxins. An IV drip would be started after exposure to the spores. The method, Fischetti reports, is already successful in mice; clinical trials will determine how long treatment must be continued, perhaps a week or so. They have also eliminated septicemia from pneumocci with the same intravenous method.

Up to now the enzymes must make contact with bacteria to kill, but Fischetti is hoping that a new generation of engineered enzymes will be able to kill pathogens inside cells too. A second disadvantage is that they are effective only against gram-positive bacteria, although that group includes many vicious pathogens.

But phage enzymes seem to offer one very big advantage: resistance to them has yet to develop. Fischetti says, “We've tried very hard to identify resistant bacteria, but so far we haven't found resistant organisms in all three of the enzymes we're working with. It appears to be a very rare event, much rarer than resistance to antibiotics.” Fischetti cautions against expecting that gladsome state to last forever, but he points out that even if widespread resistance takes the same 40 or 50 years that antibiotics required to become significantly resistant, phage enzymes could buy researchers decades for inventing other approaches.

## Antibiotics in the 21st Century

There is no shortage of ideas for unearthing new antibiotic candidates. Why are they so slow to enter medical practice? The bottleneck, researchers agree, lies in the development process of turning them into effective therapies. Several researchers blame the big pharmaceutical companies that got so big by leading the way to new drugs for battling infectious disease, but in recent years have dropped out. Fischetti complains, “These are the big companies that have the money to develop antiinfectives, but they leave it to small biotech companies, and it's not going to happen as rapidly as it should. I think it's really unconscionable for these big companies to drop the ball because it's not going to be a billion-dollar market for them and that's what they're looking for.”

Half a billion at least, says Francis Tally, a big pharmaceuticals veteran who is now chief scientific officer at Cubist Pharmaceuticals, a biotech company located in Lexington, Massachusetts. According to Tally, Cubist produced daptomycin, approved in September 2003, by licensing it from Eli Lilly, which shelved the new compound after concluding its potential market was only $250 million.

But, Tally argues, the size of the market is not the only barrier to new antibiotics. Combinatorial chemistry and the genomics revolution have simply not delivered on their early promise. “The pipeline is very dry,” he says. “There's been a real lag at the basic research level.”

“Antibiotic discovery is hard,” Shapiro says. “It's a huge long process to get a decent antibiotic.” Walsh agrees. “It's easier to find inhibitors of particular enzymes for particular processes—and a very long road to convert that into something for development.”

In the meantime, there is a rising clamor to slow down the rate at which bacteria develop resistance. Doctors are exhorted to cut back on prescribing antibiotics and decline to prescribe for viral diseases, which antibiotics can't combat, even when their patients badger them.

But even if antibiotic consumption slowed, we will still need new antibiotics. “I always say it's not a matter of if, it's only a matter of when,” says Walsh. “There will always be a need for new antibiotics because the clock starts ticking on the useful lifetime of any antibiotic once you start to use it. That cannot be argued.”

## Abbreviations

DARPA: Defense Advanced Research Projects Agency
DOD: Department of Defense
TB: tuberculosis

## References

[pbio-0020053-Artsimovitch1] Artsimovitch I, Chu C, Lynch AS, Landick R (2003). A new class of bacterial RNA polymerase inhibitor affects nucleotide addition. Science.

[pbio-0020053-Berdis1] Berdis AJ, Lee I, Coward JK, Stephens C, Wright R (1998). A cell cycle-regulated adenine DNA methyltransferase from Caulobacter crescentus processively methylates GANTC sites on hemimethylated DNA. Proc Natl Acad Sci U S A.

[pbio-0020053-Grundling1] Grundling A, Manson MD, Young R (2001). Holins kill without warning. Proc Natl Acad Sci U S A.

[pbio-0020053-Hopwood1] Hopwood DA (2004). Cracking the polyketide code. PLoS Biol.

[pbio-0020053-Kohli1] Kohli RM, Walsh CT, Burkart MD (2002). Biomimetic synthesis and optimization of cyclic peptide antibiotics. Nature.

[pbio-0020053-Loeffler1] Loeffler JM, Nelson D, Fischetti VA (2001). Rapid killing of Streptococcus pneumoniae with a bacteriophage cell wall hydrolase. Science.

[pbio-0020053-McAdams1] McAdams HH, Shapiro L (2003). A bacterial cell-cycle regulatory network operating in time and space. Science.

[pbio-0020053-McCormick1] McCormick AW, Whitney CG, Farley MM, Lynfield R, Harrison LH (2003). Geographic diversity and temporal trends of antimicrobial resistance in Streptococcus pneumoniae in the United States. Nat Med.

[pbio-0020053-Schuch1] Schuch R, Nelson D, Fischetti VA (2002). A bacteriolytic agent that detects and kills Bacillus anthracis. Nature.

[pbio-0020053-Tang1] Tang Y, Lee TS, Khosla C (2004). Engineered biosynthesis of regioselectively modified aromatic polyketides using bimodular polyketide synthases. PLoS Biol.

[pbio-0020053-Walsh1] Walsh CT (2003). Where will new antibiotics come from?. Nat Rev Microbiol.

[pbio-0020053-Watanabe1] Watanabe K, Rude MA, Walsh CT, Khosla C (2003). Engineered biosynthesis of an ansamycin polyketide precursor in Escherichia coli. Proc Natl Acad Sci U S A.

